# The microRNAs: Small size, big value,…

**DOI:** 10.4103/2152-7806.68706

**Published:** 2010-08-25

**Authors:** Ihsan Solaroglu, John H Zhang

**Affiliations:** 1 Zhang Neuroscience Research Laboratories, Loma Linda University School of Medicine, Loma Linda, CA 92350 USA; 2 Department of Neurosurgery, Ankara Atatügrk Research and Education Hospital, Ankara 06800, Turkey

MicroRNAs (miRNAs) are a class of small non-coding RNA molecules of about 22 nucleotides in length, and they regulate gene expression at the post-transcriptional level. The first miRNAs were identified in the early 1990s in *Caenorhabditis elegans* by Rosalind C. Lee, Rhonda L. Feinbaum, and Victor Ambros.[6] Since then, hundreds of miRNAs have been identified in plants and animals. Today, it is estimated that human genomes encode up to 1000 unique miRNAs and well over two-thirds of human protein coding genes appear to be conserved targets of miRNAs.[1,2,7] Although our understanding of the biogenesis and functions of miRNAs has evolved over the last few years, their physiological role remains to be explored.[3]

The transcription of miRNAs depends on their localization within the genome. The miRNAs are generated by a multistep process: the nuclear processing of the primary-miRNAs (pri-miRNA), the nuclear export of the precursors (pre-miRNA), and finally the generation of the ~22 nucleotide mature miRNA in cytosol.[9,10] Once made, by inhibiting translation or promoting mRNA degradation, or by transcriptional and translational activation, miRNAs have the ability to modulate gene expression. The miRNAs may regulate gene family by directly targeting their coding regions.

A growing body of evidence suggests that by affecting gene regulation, miRNAs are likely to be involved in almost every cellular process such as proliferation, differentiation, cell death, and the regulation of cellular development. It is becoming clear that miRNAs are essential regulators of many of the major signaling pathways implicated in these processes. However, little is known about it.

## WHAT IS MIRNAS ROLE IN THE FUTURE NEUROSURGERY?

During the past decade, the role of miRNAs in a variety of pathological processes including neuronal development, apoptosis, inflammation, and angiogenesis has been discovered. A few studies assessed the role of miRNAs in Alzheimer disease and amyotrophic lateral sclerosis. However, a significant attention has been focused on the role of miRNAs in brain tumor development and progression.

Although still not well characterized, recent evidences indicate that miRNAs can function as tumor suppressors and oncogenes, and may have a central role in tumorigenesis. For example, it is well established that both epidermal growth factor receptor (EGFR) and the phosphatidylinositol 3-kinase (PI3K)/Akt pathways play a major role in the pathogenesis of human gliomas. In glioblastoma multiforme (GBM), EGFR overexpression and/or expression of its constitutively activated variant, EGFRvIII, result in the downstream activation of the PI3K/Akt pathway. The Akt activation is correlated with the increased tumorigenicity and invasiveness in gliomas. It has been shown that miR-7 potently and directly regulates EGFR, and the Akt pathway is directly inhibited by miR-7. The Notch pathway, which also plays an important role in glioma cell survival, has been shown to be inhibited by miR-326.[4,5]

Moreover, aberrant expression of miRNAs has also been shown to be involved in tumorigenesis. EGFR amplification and phosphatase tensin homolog on chromosome ten (*PTEN*) loss are two common genetic alterations seen in gliomas. *PTEN*, a tumor suppressor gene, is lost in up to 70% of glioblastomas. Downregulation of miR-21 with a specific antisense oligonucleotide inhibits EGFR pathway and suppresses the growth of human GBM cells, independent of *PTEN* status.[11] Exploring the underlying mechanisms of miRNA action in glioma biology will provide novel therapeutic targets, and will be useful for the clinical management of this lethal and devastating neurological disorder. The miRNAs have a role not only in glioma biology, but also in medulloblastoma tumorigenesis. A recent study showed the association between cyclin dependent kinase 6 (CDK6) and miR-124 in medulloblastoma cell lines. It has been shown that miR-124 is significantly decreased in medulloblastoma cells. The re-expression of miR-124 in medulloblastoma cells decreased expression of CDK6 protein, which is known to be an important regulator of cell cycle progression.[8] It is important to note that overexpression of CDK6 has been shown to correlate significantly with poor prognosis in medulloblastoma. Moreover, there is an increasing interest in an association between miRNA expression in brain tumors and chemo- and radiosensitivity, prognosis, and invasiveness. Not surprisingly, these results may provide the grounds for new therapeutic strategies in central nervous system (CNS) tumors in the near future. However, the puzzle is waiting to be solved.

As summary, the miRNAs contribute to various cellular processes, but the detailed mechanisms remain poorly understood. A detailed knowledge of the biogenesis, functions, and targets of miRNAs would be important for a better understanding of their potential in the treatment or prevention of CNS disorders.
